# Understanding Multidisciplinary-team Practice in Developing Home Assessment Tools: A Systematic Review

**DOI:** 10.1093/geroni/igab046.2372

**Published:** 2021-12-17

**Authors:** Mengzhao Yan, Kathleen Wilber, Jon Pynoos

**Affiliations:** 1 University of Southern California, Los Angeles, California, United States; 2 University of Southern California, University of Southern California, California, United States

## Abstract

As widely used instruments to identify risk factors and lay out preliminary plans of how to improve the built environment, home assessment tools play an important role in the process of modifying homes for older adults. Developed by a variety of disciplines and tailored to meet various needs, home assessment tools focus on features in homes and how they meet or hinder an older person’s ability to accomplish tasks — in other words, person-environment fit. Based on a comprehensive review of ten evidence-based home assessment tools identified by researchers at the USC Leonard Davis School of Gerontology and the National Council on Aging, we found that a common assessment strategy is the use of multidisciplinary teams (MDTs) in developing and testing the assessment tools to ensure the reliability vs validity of different home modification programs. To understand the nature of MDT practice and derive a set of generalizable protocols for developing person-centered home assessment tools, we conducted a systematic analysis of the ten evidence-based home assessment tools and 41 peer-reviewed journal papers about how the tools were developed, used, and modified. In addition, we applied the RE-AIM framework (Reach, Effectiveness, Adoption, Implementation, and Maintenance) to examine the use of MDTs in developing the tools and carrying out the programs. Based on our analysis, we propose a set of preliminary protocols for developing home assessment tools and a logic model for conducting person-centered home modification programs.

